# Event and Comment

**Published:** 1916-04

**Authors:** 


### DENTAL REGISTER

Vol. LXX APRIL, 1916 No. 4

### EVENT AND COMMENT

NEW DENTAL SCHOOL IN NEW YORK. A new dental school is to be established as a department of the College of Physicians and Surgeons, which is a department of Columbia University. There is no doubt that a first class university school in New York will meet the general approval of the profession. It is to be conducted on different lines from any other school in existence. The plan is to require two years of academic work for admission and the studies of the first two years are to be the same as those taken by students of medicine. At the end of his second year in the course a student may continue as a medical student, or take up the proper technical and scientific studies for two years more and graduate with the dental degree. Of course a medical student at the end of his second year may switch to dentistry. By this plan a student could take both the medical and dental degrees in six years, and add the literary degree with another year of academic work probably. This plan has been discussed many times before, but this is the first effort to put it into practical effect. Such a system will bring medicine and dentistry together more definitely than any other proposition that has been suggested. There are those who think this method of instruction will not develop the best type of technical dentist. Possibly this may be true; but is the time not approaching when more pathology and less technics will be demanded of our educational institutions? There will doubtless always be a demand for rescue and repair dentistry, but at present there is a greater interest expressed in the nature of dental diseases and their influence on general health than ever before. Physicians have at last awakened to the fact that lack of dental attention and improper dentistry are alarming factors in causing systemic disorders of a most baffling nature. Therefore it seems reasonable that dental education will necessarily undergo a considerable modification as to the contents of its curriculum in the near future, to meet this increasing demand for better dental hygiene and therapeusis, and possibly for less strenuous technical operations and prosthetic procedures. Besides all this, there is a growing demand for preventive dentistry, which we have, as a profession, scarcely done more than to utilize our present knowledge of technical methods to even investigate. Necessarily this will be undertaken at an early period in the child's life, so far as we now know, or can see any way of achieving it, by medical science and dental surgery combined. Some dentists and physicians who are studying this problem believe that by the combined efforts of educated and skilled dental and medical practitioners co-operating, not only will dental caries be rarely found in children's teeth, but that many other diseases that are common to children will be brought under control. In view of this situation should we not hail this new enterprise at Columbia University as one of the most important steps ever taken for the benefit of suffering humanity, to the glory of the healing art, to say nothing of the tremendous possibilities for the health and happiness of our people? From an educational standpoint it has also many points of interest which we may not discuss here. Let us hope that those in charge of the enterprise realize that they are undertaking a most important and far reaching program, which, if it shall be adequately fostered, is bound to develop into large and consequential proportions. Let us also hope that the men in charge may be so wise as to see the tremendous importance of making every personal sacrifice which they will undoubtedly be called upon for, and that harmony and zealous co-operation may characterize their endeavors to do a grand work for humanity. There is no better place for such a school and no greater field of influence. Every thoughtful dentist will welcome this new effort and most cordially endorse the action of the College of Physicians and Surgeons in organizing this school on this ideal basis.

UNJUST DISCRIMINATION AGAINST ARMY DENTAL CORPS. "The attention of the officers and the Legislative Committee of the National Dental Association is hereby called to a bill recommended to Congress by the Bureau of Medicine and Surgery, which provides for the Army Dental Corps *'assimilated rank'* only, placing the dentist on the plane with the veterinarian, and beneath the Line and Medical officers. This is an indignity to the entire dental profession. *The Dental Corps is a part of the Medical Corps.* This discrimination should be resented and resisted with every available force." This Editorial in *Items of Interest* for March, should interest and arouse the Dental Profession to a sense of its rights with a protest to Washington.

